# 1450. Managing a Multidrug-resistant *Acinetobacter baumannii* Outbreak in the ICU: A Successful KAIZEN Program Using Process Indicators for Antimicrobial Stewardship, Hand Hygiene, and Standardization

**DOI:** 10.1093/ofid/ofad500.1287

**Published:** 2023-11-27

**Authors:** Hideki Kawamura, Syoko Arimura, Nao Murata, Akari Shigemi, Syuhei Niiyama, Junichiro Nishi, Yasuyuki Kakihana

**Affiliations:** Kagoshima University Hospital, Kagoshima, Kagoshima, Japan; Kagoshima University Hospital, Kagoshima, Kagoshima, Japan; Kagoshima University Hospital, Kagoshima, Kagoshima, Japan; Kagoshima University Hospital, Kagoshima, Kagoshima, Japan; Kagoshima University Hospital, Kagoshima, Kagoshima, Japan; Kagoshima University Hospital, Kagoshima, Kagoshima, Japan; Kagoshima University Hospital, Kagoshima, Kagoshima, Japan

## Abstract

**Background:**

Between 2016 and 2018, we detected Multidrug-resistant *Acinetobacter baumannii* (MDRA) carrying the IMP-1 gene in 15 patients including 14 of whom had been admitted to the ICU (Figure 1). The hospital environment including bed rail and mattresses became a reservoir for MDRA. We implemented the KAIZEN program in the ICU, which included process indicators for antimicrobial stewardship (AS), hand hygiene, and standardizing. The aim of this study is to evaluate the effectiveness of this KAIZEN program and establish infection control measures against MDRA.

**Figure 1.**
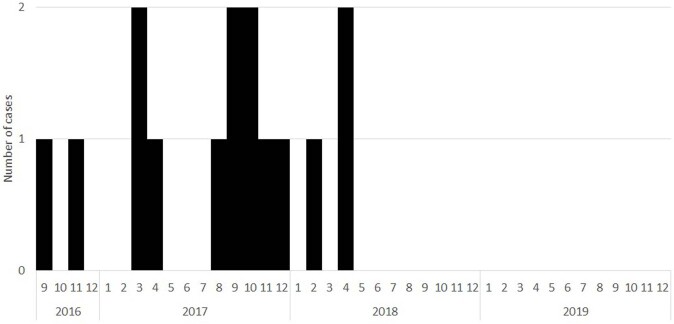
Epicurve of Multidrug-resistant Acinetobacter baumannii

**Methods:**

In August 2018, we implemented several process indicators in the ICU at August 2018 to monitor antimicrobial use density (AUD) for carbapenems (target: less than 10 per 100 patient-days), use of an alcohol antiseptic agent (target: more than 200 times per patient-days), and weekly infection control rounds. We implemented a policy requiring staff to disinfect their hands when entering and exiting patient rooms, displayed posters reinforcing the rules of hand hygiene, used video monitoring to provide feedback on compliance rates, and encouraged staff to remind each other. Doctors were required to obtain permission using a checklist before prescribing carbapenems and held weekly conferences with ICU doctors to review AS bundles. We formed a 5S (Sorting, Setting-in-order, Shining, Standardizing, Sustaining) working group to discuss and implement strategies to maintain a clean environment and address infection control issues raised in the weekly rounds

**Results:**

AUD for carbapenems decreased from 17.8 in 2016 to 6.7 in 2019, while use of an alcohol antiseptic agent increased from 51.5 in 2016 to 219.6 in 2019 (Figure 2). There was improvement in the issues identified during infection control rounds related to 5S, as demonstrated by a reduction in the number of previously identified problems (Figure 3). The MDRA outbreak was deemed successfully resolved based on improvements in process indicators and the absence of MDRA cases in patients or the environment for a year.

**Figure 2.**
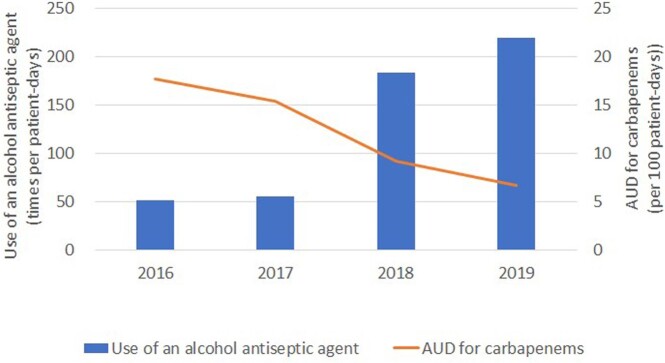
Antimicrobial use density (AUD) for carbapenems and Use of an alcohol antiseptic agent from 2016 to 2019 in ICU

**Figure 3.**
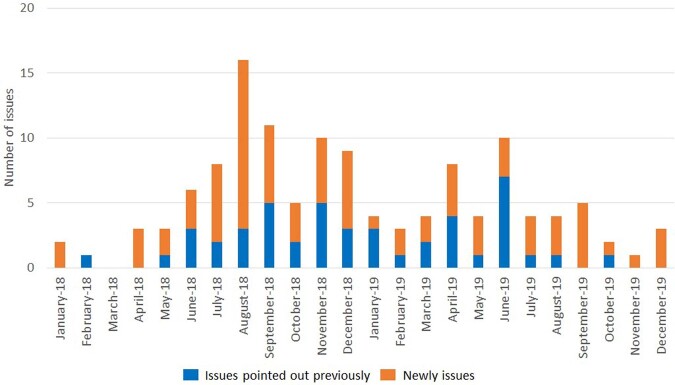
The number of issues about 5S infection control rounds

**Conclusion:**

The KAIZEN program, which involved planning and implementing improvement measures based on process indicators, effectively controlled the MDRA outbreak by standardizing infection control and antimicrobial use through the establishment of rules.

**Disclosures:**

**All Authors**: No reported disclosures

